# The effects of family-centered empowerment model on depression, anxiety, and stress of the family caregivers of patients with COVID-19: a randomized clinical trial

**DOI:** 10.1186/s12875-022-01795-8

**Published:** 2022-07-26

**Authors:** Mohammad Namazi Nia, Samira Mohajer, Nasser Bagheri, Tahere Sarboozi-hoseinabadi

**Affiliations:** 1 Department of Nursing, School of Nursing and Midwifery, Torbat Heydariyeh University of Medical Sciences, Torbat Heydariyeh, Iran; 2 Health Sciences Research Center, Torbat Heydariyeh University of Medical Sciences, Torbat Heydariyeh, Iran; 3 Nursing and Midwifery Care Research Center, Mashhad University of Medical Sciences, Mashhad, Iran; 4 Department of Nursing Science, Faculty of Medicine, University of Malaya, Kuala Lumpur, Malaysia; 5 Visual and Decision Analytics (VIDEA) Lab, Health Research Institute, University of Canberra, Canberra, Australia; 6 The National Centre for Epidemiology and Population Health, College of Health and Medicine, The Australian National University, Canberra, Australia

**Keywords:** Family Caregivers, Depression, Anxiety, Stress, Family-Centered Empowerment Model, COVID-19, Randomized Control Trial

## Abstract

**Background:**

Taking care of patients with Covid-19 is regarded as a challenging task for family caregivers. Hence, a Family-Centered Empowerment Model (FCEM) should help them achieve greater psychological strength throughout the home healthcare process.

**Methods:**

This study is a randomized clinical trial with two groups; besides, pre-test and post-test designs were conducted based on the CONSORT checklist from April to July 2020, in Iran. Seventy family caregivers were randomly assigned to FCEM (*n* = 35) and control (*n* = 35) groups. Then, four stages of FCEM in four online sessions were provided to the participants of the intervention group via WhatsApp messenger. The procedure started at the patient's discharge and continued for two weeks. The demographic information questionnaire and Depression Anxiety Stress Scale (DASS-21) were employed before and five days after the FCEM sessions to gather the required data.

**Results:**

The sample was made up of 55.8% women and 44.2% men caregivers, with a mean age of 42.5 years. The results demonstrated a substantial difference in the average score of stress (*p* = 0.023), anxiety (*p* = 0.003), and depression (*p* = 0.012).

**Conclusions:**

The combination of a face-to-face orientation session and online methods of FCEM is likely to lower stress, anxiety, and depression in family caregivers, which can be contributed to the practicability, simplicity, and effectiveness of this home health intervention.

**Trial registration:**

This study (no. IRCT20180429039463N2) was registered in the Iranian Registry of Clinical Trials on 10/04/2020.

**Supplementary Information:**

The online version contains supplementary material available at 10.1186/s12875-022-01795-8.

## Background

The novel coronavirus pandemic, COVID-19, started in Wuhan, China on December 31, 2019 [1], and World Health Organization (WHO) declared the Coronavirus epidemic as a Public Health Emergency of International Concern (PHEIC) on January 30, 2020 [2]. According to WHO, about 544,544,105 infected cases and 6,341,231 deaths were reported in more than 228 countries from December 2019 to June 2022. At the same time, the number of confirmed cases and deaths in Iran were 7,234,988 and 141,366, respectively [3]. Mass COVID-19 vaccination rollout is a public health top priority to mitigate the pandemic. However, refusal of COVID-19 vaccination could prolong the battle against this pandemic and result in needless suffering and death [4].

Moreover, the COVID-19 pandemic has had a significant impact on public mental health [5], which led to psychological issues such as stress, anxiety, and depression among communities [5–7]. Limited knowledge and excessive media exposure about the confirmed cases and the number of deaths may cause social anxiety and fear to patients and their family members [8]. The result of studies in Iran revealed that negative emotions such as fear, depression, and stigma are associated with SARS-CoV-2 infection, which also lead to moderate to severe anxiety [6, 7]. Besides, the lack of any definitive treatment or prevention has caused a lot of stress, anxiety, and depression in communities [9]. Thus, the Iranian Government, like other countries, strives to raise the public's awareness of the truth of the pandemic by providing daily updates about surveillance and active cases on websites and social media as well as implementing interventional self-care strategies so that they can cope with this crisis efficiently. It is also noteworthy that the hospitalization of patients with COVID-19 is a critical stressor for family members [10]. The findings of a seminal study by Anderson et al. (2020) revealed that patients with COVID-19 put both themselves and their family members under a lot of stress, which is doubled when the patient is admitted to the hospital [9].

Of notable importance, the growing prevalence of COVID-19 and the lack of hospital facilities have contributed to the early discharge of COVID-19 patients from the hospital. Consequently, when family members move into their caregiving roles, they may experience a collection of positive and negative situations [10]. According to the principal conceptual model of caregiving, the patients and their caregivers are likely to experience significant concerns both at the initial stage and continuation of such psychological problems [11]. Given the religious and cultural values as well as the strong family relationships, Iranian family caregivers, who are often women, are willing to take care of the patients and provide a comprehensive support network to them. They are often exposed to physical and psychological needs, such as caregiving overload and emotional stress related to the home healthcare process; furthermore, they would be expected to reduce their work time, social activities, and relationships with family and friends. However, the COVID-19 conditions have overwhelmed family caregivers [10]. Hence, caregivers’ practical stressors cause psychological stress, state-trait anxiety, and situational depression because of their challenging responsibilities [11].

Anxiety is a common emotional response to a perceived threat [12]. The anxiety of family caregivers limits their capacity to help the patient and can also exacerbate the patient's anxiety [13]. Nonetheless, home caregivers of the COVID-19 patients are experiencing greater levels of stress, anxiety, and depression symptoms [14, 15] for a variety of reasons, including the fear of infection or transmitting it to others, poor community and governmental support, lack of access to medical care, and stigma [16]. These family caregivers would also face critical challenges compared to other caregivers because of the limited training and resources at hand as well as their insufficient knowledge about this emergent disease [10]. Therefore, psychobehavioural interventions are needed to facilitate the management and control of the psychological issues related to the COVID-19 outbreak [7].

Several behavioral theories and psychological counseling interventions, such as hope building, reassurance, health belief model, self-management, and guidance techniques have been proposed to reduce psychological distress and improve the mental health of family caregivers [17–22]. It seems that current interventional activities are insufficient, and future home healthcare interventional strategies should be focused on enhancing family empowerment and resilience as well as improving quality of life and mental wellbeing [23–25]. Similarly, educational interventions are helpful strategies to empower communities encountering the threat of pandemics like Covid-19. The results of another study by Zahedifar et al. (2021) in Iran showed that educational intervention could reduce the anxiety of patients referred to medical centers during the Covid-19 pandemic. In addition, such strategies can empower the public and diminish the negative emotional effects of the pandemic, help people cope with the existing situation, and decrease the risk of suffering future psychological disorders [26]. Despite performing diverse national and international studies, there have been no studies focusing on using a comprehensive program that can address all the aspects and problems of family caregivers of COVID-19 patients as a backbone of patient-support care. An empowerment program improves self-control and preventive behaviors [27]. Nurses are well-positioned to identify, assess, and intervene in the management of anxiety, stress, and depression among family caregivers [12]. Thus, it is required to apply effective nurse-led empowerment programs to increase consciousness and raise awareness and self-efficacy in family caregivers. The empowerment program can be used to promote family caregivers' positive adaptation and help caregivers cope with their problems effectively, which may lead to the improvement of their mental health distress such as stress, anxiety, and depression [28].

The Family-Centered Empowerment Model (FCEM) is the most common method to identify the physical and psychological concerns of the families as well as their immediate needs. FCEM has been developed to reflect on the patients’ and their family members' impact on the following capabilities: self-problem characteristics (e.g., perspective, perceived knowledge, and perceived threat), psychological traits (self-confidence, self-reliance, and self-control), and motivational skills [29]. Tallman et al. (2012) reported that family-centered care could reduce depression, nervousness, and stress in family caregivers of older individuals with dementia [30]. By highlighting the management of home healthcare plans, this model can help decrease the healthcare inconveniences and facilitate caregivers’ role and effectiveness. The achievement of an empowerment program improves self-control and the adoption of preventive behaviors [27]. Few studies have been conducted on the effectiveness of FCEM among family caregivers of different patients in Iran [27, 31, 32]. However, no study has been conducted concerning the effectiveness of FCEM on family caregiving of patients with COVID-19. Most of the studies have focused on the experiences of patients or health care staff. Hence, caregiving should be investigated based on the family-centered empowerment model [26].

On the other hand, the COVID-19 pandemic and related restrictions led to a rapid shift towards a remote home healthcare focus in the early months of the pandemic [33]. The coronavirus disease 2019 pandemic has accelerated the process of implementing this interventional innovation internationally [33–36]. Mirhosseini et al. (2021) in Iran evaluated the effect of online psycho-education on the caring burden of the family caregivers of individuals with COVID-19; they found that online psycho-education could reduce the caring burden of these caregivers [37]. Nevertheless, there is a significant gap in the relevant literature. To bridge this gap, the present study aimed to examine the influence of FCEM (face-to-face orientation sessions and online sessions) on depression, anxiety, and stress among family caregivers of patients with COVID-19 in Iran.

## Methods

### Setting and study design

**Fig. 1:**
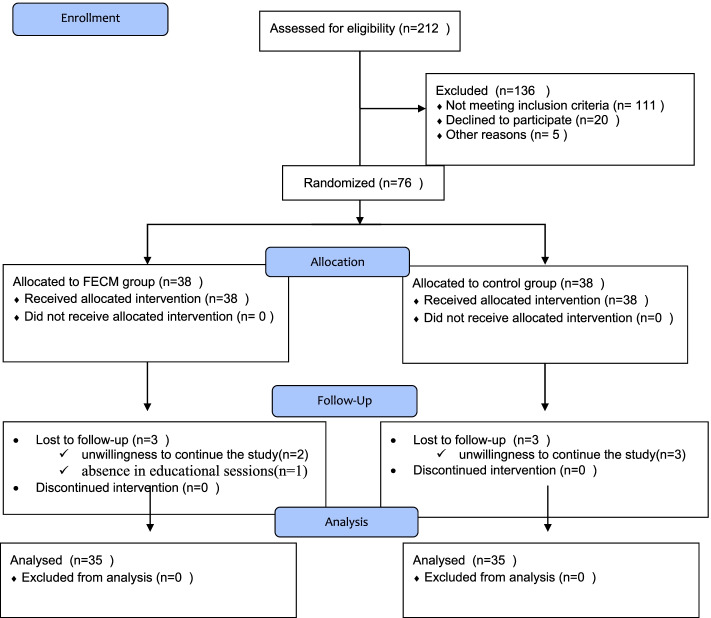
The CONSORT flow chart of study participants

This research is a randomized clinical trial with two groups; besides, pre-test and post-test designs were conducted based on the CONSORT checklist (Fig. 1). For this purpose, the Consolidated Standards of Reporting Trials checklist was employed to report the study findings (S1, CONSORT checklist).

This study aimed to investigate the effects of FCEM on depression, anxiety, and stress among the family caregivers of patients with COVID-19. The four-stage FCEM was provided to the intervention group via WhatsApp Messenger in four online sessions, which started from the patient's discharge and continued for duration of two weeks. Consequently, the demographic information questionnaire and Depression Anxiety Stress Scale-21 were employed to collect the data before the study and also five days after the FCEM sessions.

### Populations, inclusion, and exclusion criteria

The statistical population includes 70 family caregivers from living in Torbat-e-Heydariyeh, Iran, who participated in the study from April to July 2020. The following inclusion criteria were taken into account for this study: for patients include patients with COVID-19 who were treated and discharged, the age range between 18 and 60 years old, and no record of underlying diseases other than COVID-19. Exclusion criteria for the patients include death, diagnosis of other diseases, and readmission. The following inclusion criteria were used for family caregivers: family member providing care for a patient with COVID-19, family caregiver’s access to communication devices such as mobile phones, personal computers, and tablets equipped with WhatsApp Messenger, reading and writing skills literacy, and no record of working as a member of a healthcare team. Exclusion criteria for family caregivers include the age range of fewer than 18 and beyond 60 years, absence in more than one educational session, and reluctance to continue the study.

### Measurement instruments

Data collection instruments include a six-item demographic information questionnaire (age, sex, marital status, living place, patient-caregiver relationship, and education) and the DASS-21 to assess depression, anxiety, and stress among family caregivers of patients with COVID-19. This questionnaire was first presented by Lovibond PF and Lovibond SH in 1995 [38]. The 21-item questionnaire was evaluated based on the Likert scale, and each of the three scales contains seven items. The Stress section assesses fear, shortness of breath, tremors, dry mouth, palpitation, difficulty in starting work, and difficulty in relaxing. The Anxiety section evaluates autonomic arousal, situational anxiety, skeletal muscle effects, and subjective experience of apprehension distress. Finally, the Depression section measures self-depreciation, life devaluation, hopelessness, dysphoria, and the feeling of indifference/sedentary [32].Two research team members (Mehdipour and Najafi) reviewed and approved the validity and reliability of the DASS-21 scale [39, 40]. Besides, the content quality validity method was used to determine the validity of the DASS-21. The questionnaire was provided to 10 faculty members of Torbat-e-Heydariyeh University of Medical Sciences for further evaluation, and the ultimate instrument was used after the necessary modifications were made. To confirm the reliability (internal consistency) of the study, we tested the DASS-21 scale on 10 subjects, and the obtained Cronbach's alpha coefficient was 0.81. The total score of each DASS-21 subscale should be obtained through the seven respective item scores. Each item score ranges from zero (did not apply to me at all) to three (applied to me very much). Since this questionnaire is a short form of the original version (42 items), the final score of each subscale must be doubled. The DASS-21 and the demographic information questionnaire were prepared online, and the link was sent electronically to the family caregivers of patients with COVID-19 via WhatsApp Messenger for the measurement of pre-test as the baseline data.

### Sample size and randomization

The necessary sample size was estimated according to the findings of a pilot study on 20 family caregivers (10 individuals in each group), with a 95 percent confidence interval and 80 percent test power. Thirty-four family caregivers were included in each group using the following formula:

$$n=\frac{(\mathrm Z1-{\displaystyle\frac{\mathrm\alpha}2}+\;\mathrm Z1-\mathrm\beta2)\;(\mathrm S12+\mathrm S22)}{\left(X1-X2\right)2}$$

According to the CONSORT flow diagram of this study, about two-thirds of a total number of 212 family caregivers assessed for eligibility did not meet the inclusion criteria Given a 10-percent dropout possibility, 38 family caregivers were assigned to each group. Then, three family caregivers were excluded from the intervention group (two family caregivers due to reluctance to continue the study and one due to absence in educational sessions) and three family caregivers were excluded from the control group (due to reluctance to resume the study). Finally, 35 family caregiversies were examined in each group.

Initially, the caregivers were randomly assigned to the control and intervention groups. Eligible patients were divided into two groups of intervention or control based on a random sequence of letters A and B, which were generated by SPSS software written on small cards and retained in a sealed envelope. Then, one card would be picked out of the envelope at the beginning of each week to decide if we should conduct the intervention during that week or it is the time for the control group. Accordingly, all eligible inpatients would be allocated to either the control or intervention group. Furthermore, this approach would rule out the possibility of information disclosure among those patients who are accidentally on the ward.

### Pre-intervention

Regarding the intervention group, the researcher held an introductory face-to-face meeting with family caregivers at the patient's discharge to the COVID-19 health clinic of the hospital. First, online FCEM sessions were settled based on the participants’ agreements. Then, the priorities, educational needs, and skills required to take care of the patient with COVID-19 were assessed against a checklist. The caregivers' educational needs and problems were collected as follows: the diagnosis of the disease, personal hygiene, methods of prevention of infection transmission, medication regimen, nutrition and excretion, activities daily life, mental and psychological issues. After reviewing the needs and necessary skills, the researcher designed the educational content according to the literature as well as the ideas of the research team and infectious disease specialists of the COVID-19-health clinic.

### Intervention

The intervention program was based on the Family-Centered Empowerment Model (FCEM) and consisted of four steps, including perceived threat, problem-solving, educational participation, and evaluation, in four training sessions [29]. At first, the educational content of the online sessions was designed through a checklist based on the needs of the research units. Then, the FCEM was presented as online group discussions in five groups of seven individuals. The content was presented in four sessions (40–60 min) via WhatsApp Messenger and other applications used for this purpose. The FCEM was implemented for two weeks as follows:

- Step one (perceived threat): the researcher used two online 45-min sessions to train patients and their primary caregivers. The purpose was to increase knowledge and perceive the threat through awareness of the nature of the disease, quarantine, medication, prevention of disease transmission, as well as other significant factors, such as nutrition, bathing, disinfection and disease control.
- Step two (problem-solving): the aim of this step was skill acquisition and self-efficiency. The researcher formed an online group discussion, containing primary caregivers of the COVID-19 patients, and performed this step in two 45-min sessions. Participants also used the experiences to control the disease more effectively, become familiar with the problems and the problem-solving process, present their suggested solutions, select the best solution for the better management of the problem, and improve their skills and self-efficacy.
- Step three (educational participation): at first, the content of the online sessions were transferred to the patient and other family members. Then, the materials were shared and exchanged. At the end of the online sessions, the researcher conducted a virtual content analysis approved by an infectious disease specialist.
- Step four (evaluation): the researcher assessed all the participants in the intervention group by asking questions about the content as well as the issues discussed during the second and third sessions. Finally, the researcher resolved the remaining ambiguities and discussed the nature of the disease and the proposed care in each session to ensure that the participants have learned the content of the previous sessions comprehensively (Supplementary material 1).

### Post-intervention

The intervention and control groups were evaluated five days after the FCEM sessions. The researcher would make a telephone call to the family caregivers as a reminder and discuss any potential issues. The link to the DASS-21 would be resent to the caregivers electronically.

### Control

The patients in the control group would only experience face-to-face training (recommendation for medication and quarantine) during discharge; however, they could only use the pamphlets available in the ward, which were the same for the intervention group. All educational contents of the FCEM sessions were provided to the control group after the research.

### Statistical analysis

The obtained information was statistically analyzed using SPSS version 16. Descriptive statistics (frequency distribution, mean, and standard deviation) were employed to describe and categorize the data. On the other hand, the researchers used inferential statistics, including Chi-square, independent t-test, and Mann–Whitney tests were used to assess the hypothesis. Finally, the Kolmogorov–Smirnov test was implemented to examine the normal distribution of quantitative variables. The 95% confidence level and the significance level of 0.05 were considered for all the tests.

## Results

The majority of the family caregivers in the intervention (*N* = 20, 57.1%) and control (*N* = 19, 54.3%) groups were female. The majority of family caregivers in the intervention (*N* = 26, 74.3%) and control (*N* = 24, 68.6%) groups were married. Moreover, the patients’ children acted as family caregivers in 62.9% and 65.7% of the cases in the intervention and control groups, respectively. The results of Chi-square, Mann–Whitney, Fisher’s, and independent t-tests showed that the two groups were homogenous in terms of age, sex, education, marital status, living place, and caregiver-patient relationship (*p* > 0.05) (Table 1).

**Table 1:** Demographic variables of the caregivers of patients with COVID-19

Variable		Group		*P* value
		Intervention	Control	
Age (mean ± SD)		43.2 ± 13.2	41.8 ± 2.0	*P* = 0.641****
Sex (percent/number)	Male	15 (42.9)	16 (45.7)	*P* = 0.810*
	Female	20 (57.1)	19 (54.3)	
Education (percent/number)	Middle/high school	7 (20.0)	9 (25.7)	*P* = 0.878**
	Diploma	19 (54.2)	16 (45.7)	
	Academic	9 (25.7)	10 (28.6)	
Patient-caregiver relationship	Wife/husband	11(31.4)	9 (25.7)	*P* = 0.810*
	Children	32 (62.9)	23 (65.7)	
	Others	2 (5.7)	3 (8.6)	
Marital status (percent/number)	Single	8 (22.9)	9 (25.7)	*P* = 0.790*
	Married	26 (74.3)	24 (68.6)	
	Others	1 (2.9)	2 (5.7)	
Caregiver’s living place	City	32 (91.4)	31 (88.6)	*P* = 1.000***
	Village	3 (8.6)	4 (11.4)	

^*^ Chi-square

^**^ Mann–Whitney

^***^ Fisher

^****^ Independent-t test

In addition, it should be noted that the mean depression scores of the caregivers in the intervention and control groups were not statistically significant in the pre-test stage (*P* = 0.308). In the post-test stage, however, the mean score of depression in the intervention group (8.6 ± 3.8) was significantly lower than that of the control group (12.1 ± 7.0) (*P* = 0.012) (Table 2).

**Table 2:** Mean and standard deviation of depression, anxiety, and stress scores of caregivers of patients with COVID—19 in two groups of intervention and control

Variable		Group				*p* value
		Intervention		Control		
		Mean ± SD	No	Mean ± SD	No	
Depression	Pretest	12.2 ± 4.8	35	13.8 ± 7.6	35	*P* = 0.308*
	Posttest	8.6 ± 3.8	35	12.1 ± 7.0	35	*P* = 0.012*
Anxiety	Pretest	11.6 ± 3.2	35	12.5 ± 6.8	35	*P* = 0.476*
	Posttest	7.6 ± 2.7	35	11.2 ± 6.3	35	*P* = 0.003*
Stress	Pretest	16.0 ± 6.2	35	15.1 ± 7.4	35	*P* = 0.581*
	Posttest	10.5 ± 4.4	35	13.8 ± 7.0	35	*P* = 0.023*

^*^ Independent-t test

In the pre-test stage, there was no significant difference between the mean scores of anxiety among the family caregivers in the intervention and control groups (*P* = 0.476); nevertheless, the mean score of anxiety in the intervention group (7.6 ± 2.7) was significantly lower than the control group (11.2 ± 6.3) (*P* = 0.003) in the post-test stage (Table 2).

Although the mean scores of family caregivers’ stress were not significantly different (*P* = 0.581) between the intervention and control groups in the pre-test stage, the mean score of stress was considerably lower in the intervention group (10.5 ± 4.4) compared to the control group (13.8 ± 7.0) (*P* = 0.023) in the post-test stage (Table 2).

## Discussion

The present study aimed to investigate the contribution of FCEM to depression, anxiety, and stress in family caregivers of patients with COVID-19. According to the findings, depression, anxiety, and stress scores of these family caregivers decreased by 41, 51, and 52 points, respectively. Some studies investigated FCME among family caregivers of patients with different diseases in Iran [27, 29, 31]; however, we did not find a similar study in terms of the effects of FCEM on depression, anxiety, and stress among these participants. Hence, we decided to use the findings of other studies in this regard.

Our result showed that the depression score decreased by 41 points in the intervention group, which is in line with the results of the other studies indicating the improvement in depression level of family caregivers. Another study by Wei et al. (2020) reported that online intervention could reduce the symptoms of depression in patients with COVID-19 [35]. In addition, Daveson et al. (2014) indicated that the psycho-behavioral educational program could reduce the depression of family caregivers of patients admitted to the intensive care unit [18]. The results of a seminal study by Shatri et al. (2021) showed that the management of psychotherapy through tele-consultation or internet-based intervention on COVID-19 patients could help relieve the patient's depression symptoms [36]. In a similar vein, Sotoudeh et al. (2020) asserted that the depression of patients with Covid-19 decreased after holding four sessions of psychological counseling [17]. These results may be contributed to the fact that online interventions are complementary to face-to-face counseling. In addition, people are encouraged to use such home healthcare interventions more often because of their flexibility and applicability, independence of time and space, better protection of privacy, and lower costs.

Moreover, Aminizadeh et al. (2015) reported that e-learning would be a successful and efficient method if the content is compiled and evaluated properly [41]. We embedded the FCEM approach in online group sessions via WhatsApp Messenger for patients and their family caregivers. The online educational sessions were conducted by the nurse researcher who was responsible to educate family caregivers during the discharge phase and follow up with these patients. Since nurses are required to support and help families coping with crises in pandemics through primary contacts with patients and their families [42], their support would be vital to families to combat the inevitable consequences of the COVID pandemic, such as psychological distress. Consequently, it may be necessary to conduct such interventions, including education and family training, to mediate the pressures on caregivers and to improve the mental and physical health status among the patients and their family caregivers [25]

Our findings revealed that anxiety scores decreased by 51 points, among these family caregivers. It should be noted that our findings are consistent with the results of other studies [17, 20, 21, 27]. Moreover, the results of another study by Zahedifar et al. (2021) in Iran showed that educational intervention could help reduce the anxiety of patients referred to medical centers during the Covid-19 pandemic [26]. Their findings indicated that educational intervention could increase the awareness, perceptions, and skills; it can also facilitate the treatment and caregiving process for the caregivers because of their knowledge of different dimensions and problems of the disease and the acquisition of practical home healthcare skills. Therefore, training the patients and their families increases the patient’s awareness and reduces anxiety [27]. Besides, Chien et al. (2006) reported that a family-centered program could significantly reduce the family members’ anxiety [19].

In our study, the family-centered program was continuous, and families were contacted by telephone at least once a day in addition to face-to-face education. We also made post-discharge and follow-up telephone calls, which had a critical role in the reduction of anxiety among patients' family caregivers. Furthermore, the results of the study by Salari et al. (2020) showed that giving people the information they need about Covid-19 can increase their awareness, which ultimately leads to the reduction of anxiety [5]. It is noteworthy that caregivers often perceive they lack enough knowledge to provide appropriate care; consequently, they may experience a lot of anxiety [32]. The results of another study by Ahmad et al. (2021) showed that social media and counseling support services play an essential role in reducing anxiety among the general public. People in the community will receive daily updates on Covid-19 through television and press conferences [43]. Similarly, we implemented online group meetings via WhatsApp Messenger to increase caregivers’ awareness in the present study. The results of the aforementioned studies relatively support our findings.

The last measurement variable was stress scores of family caregivers of patients with COVID-19, which decreased by 52 points in the intervention group. Holmes et al. (2020) also found that digital interventions could reduce the level of stress in patients with COVID-19 [34]. Besides, Zhou et al. (2020) demonstrated that psychological interventions via the internet and telephone were effective in reducing the psychological burden of the public in China [44]. It seems that making phone calls to the patient's family can be effective in strengthening and increasing their knowledge capacity since families will have opportunities to discuss the potential issues with researchers during this telephone conversation [45]. Moreover, the results of another study by Jaywant et al. (2021) showed that behavioral interventions, including helping patients throughout discharge, referral to health care centers, and psychological training, could significantly reduce stress in patients [21]. We implemented face-to-face and online sessions of FCEM intervention at discharge and after two weeks of recovery from COVID-19 because stress management is critical for family caregivers during this time. It should be noted that our findings are consistent with the results of these studies.

In brief, the rate of depression, anxiety, and stress reduced in the control group at the end of the study, which was also lower compared to the intervention group. The reason may be that macro-policies related to adherence to health protocols, including public education through the national media, were adopted during the COVID-19 epidemic to reduce the prevalence of the disease in the world, especially in Iran. Therefore, the public awareness of the disease, appropriate caring methods, and adaptation have increased accordingly. However, the findings highlighted the significance of raising knowledge, awareness, and problem-solving management in dealing with patients' problems. Moreover, due to the religious and cultural values as well as the strong family relationships, Iranian family caregivers, who were mainly female in the present study, are willing to take care of the patients and provide a comprehensive support network to them. Therefore, FCEM enabled the family caregivers to cope with the stressors more efficiently so that they could tolerate the psychological, physical, and economic effects of home healthcare through participatory planning. Our results confirmed the findings from different settings and populations. Nonetheless, some differences are witnessed due to disparate service utilization, cultural diversity, and styles of communication and coping.

### Strength and limitations

The present study represented the first attempt to use FCEM with the combination of a face-to-face orientation session and online methods in depression, anxiety, and stress of family caregivers of patients with COVID-19. Various factors seem to be effective in the mental health status of caregivers of patients with COVID-19. One of the limitations of this study was the different learning levels of families and the transfer of education to patients. Furthermore, the basic level of the mental health status of caregivers. In addition, the hospital ward (routine care or ICU) and the length of hospitalization may affect the mental health of caregivers. Besides, the intervention lasted only two weeks because of the nature of COVID-19 disease (incubation period, illness, and recovery), so more interventions may be needed for more stable changes. Given that it was not possible to do sampling in other cities in Iran, there may be a bias in the completion of the electronic questionnaires. Therefore, another limitation of the study was the follow-up gap for some cases in order to evaluate the effectiveness of the intervention until sometime after the end of the intervention. This can be an integral variable given the pandemic and the possibility of infection in other family members.

## Conclusion

The results of the present study showed that the combination of a face-to-face orientation session and online methods of FCEM could reduce the depression, anxiety, and stress scores among family caregivers of patients with COVID-19. Such interventions seem to help these family caregivers increase the quality of  home healthcare because they are efficient, inexpensive, and safe. Nonetheless, it is essential to conduct further studies to examine the mechanism of the influence of FCEM on depression, anxiety, and stress. Therefore, online FCEM is suggested to be employed for other diseases in further research, particularly during the limitations of the current pandemic and difficulties in performing educational courses. However, the duration of the FCEM intervention should be increased to obtain more accurate results.

## Supplementary Information

**Additional file 1:**Steps of the family-centered empowerment model.

## Data Availability

All data generated or analyzed during this study are included in this published article (including the supplementary information files).

## Abbreviations

FCEM: Family-centered empowerment model
WHO: World Health Organization
PHEIC: Public Health Emergency of International Concern
DASS-21: Depression, Anxiety and Stress Scale
