# Supplementary material for: The effects of family-centered empowerment model on depression, anxiety, and stress of the family caregivers of patients with COVID-19: a randomized clinical trial
Source: BMC Prim Care. 2022 Jul 26;23:185. doi: 10.1186/s12875-022-01795-8 (PMC9315845; doi:10.1186/s12875-022-01795-8)
Supplement: Supplementary file 1 — Additional file 1:Steps of the family-centered empowerment model. [file 12875_2022_1795_MOESM1_ESM.docx]

Supplementary material

**Steps of the family-centered empowerment model**

| Steps | Session content | Aim |
| --- | --- | --- |
| 1  (perceived threat) | the researcher used two online 45-minute sessions to train patients and their primary caregivers. | increase knowledge and perceive the threat through awareness of the disease's nature, quarantine, drug use, prevention of disease transmission, and important issues regarding nutrition, bathing, disinfection, and other factors affecting disease control. |
| 2  (problem-solving) | The researcher formed an online group discussion, containing primary caregivers of the COVID-19 patients and performed this step in two 45-minute sessions. Participants also used the experiences to better control the disease, and become familiar with the problems and the problem-solving process, presented their suggested solutions, and then selected the best solution for the better management of the problem and to improving their skills and self-efficacy. | skill and self-efficiency acquisition. |
| 3  (educational participation) | the materials discussed in the online sessions were transferred to the patient and other family members by an active family member. At the end of the online sessions, the researcher conducted virtual content approved by an infectious disease specialist. | share and exchange the educational materials |
| 4  (evaluation) | the researcher assessed all the participants in the intervention group by asking questions about the whole content taught, and the issues discussed during the second and third sessions. | the researcher resolved remaining ambiguities, discussed the disease and the care taken in each session to ensure that the contents of the previous sessions were learned. |
